# Depression of oncogenecity by dephosphorylating and degrading BCR-ABL

**DOI:** 10.18632/oncotarget.13754

**Published:** 2016-12-01

**Authors:** Miao Gao, Zheng-Lan Huang, Kun Tao, Qing Xiao, Xin Wang, Wei-Xi Cao, Min Xu, Jing Hu, Wen-Li Feng

**Affiliations:** ^1^ Department of Clinical Hematology, Key Laboratory of Laboratory Medical Diagnostics Designated by The Ministry of Education, Chongqing Medical University, Chongqing, People's Republic of China; ^2^ Department of Immunology, Molecular Medicine and Cancer Research, Chongqing Medical University, Chongqing, People's Republic of China; ^3^ Department of Hematology, The First Affiliated Hospital, Chongqing Medical University, Chongqing, People's Republic of China

**Keywords:** chronic myeloid leukemia, BCR-ABL, Y177, protein tyrosine phosphatase, ornithine decarboxylase

## Abstract

Aberrant phosphorylation and overexpression of BCR-ABL fusion protein are responsible for the main pathogenesis in chronic myeloid leukemia (CML). Phosphorylated BCR-ABL Y177 recruits GRB2 adaptor and triggers leukemic RAS-MAPK and PI3K-AKT signals. In this study, we engineered a SPOA system to dephosphorylate and degrade BCR-ABL by targeting BCR-ABL Y177. We tested its effect on BCR-ABL phosphorylation and expression, as well as cell proliferation and apoptosis in CML cells. We found that SPOA remarkably dephosphorylated BCR-ABL Y177, prevented GRB2 recruitment, and uncoupled RAS-MAPK and PI3K-AKT signals. Meanwhile, SPOA degraded BCR-ABL oncoprotein in ubiquitin-independent manner and depressed the signal transduction of STAT5 and CRKL by BCR-ABL. Furthermore, SPOA inhibited proliferation and induced apoptosis in CML cells and depressed the oncogenecity of K562 cells in mice. These results provide evidence that dephosphorylating and degrading oncogenic BCR-ABL offer an alternative CML therapy.

## INTRODUCTION

Chronic myeloid leukemia (CML) is resulted from t (9; 22) (q34; q11) chromosome translocation, which produces a bcr-abl fusion gene on the Philadelphia (Ph) chromosome, encoding p210^BCR-ABL^ fusion protein with aberrant tyrosine kinase activity [1, 2]. This constitutively activated tyrosine kinase activates multiple signal transduction pathways including RAS-MAPK, PI3K-AKT, STAT5, and CRKL, which leads to malignant proliferation and decreased apoptosis of cells [3, 4]. Consequently, BCR-ABL tyrosine kinase has been considered as the most important target for CML treatment. Imatinib (Gleevec, STI571), one of the BCR-ABL tyrosine kinase inhibitors (TKIs), has been recommended as the first-line treatment for CML, which competitively inhibits the binding of adenosine triphosphate (ATP) to BCR-ABL kinase domain and blocks the substrate phosphorylation of BCR-ABL [5]. Although ~80% of newly diagnosed chronic-phase CML patients receiving imatinib acquire complete cytogenetic response, imatinib resistance is inevitable in ~40% of patients attributed to BCR-ABL kinase domain mutations [6]. The second generation of TKIs, such as dasatinib and nilotinib, are potent against most of the mutations except T315I, which prevents TKIs from binding to BCR-ABL kinase domain [7]. So, alternative strategies are needed to overcome TKIs resistance.

Given the fact that mutations in ABL kinase domain contribute to resistance to imatinib, we make efforts to explore a target outside ABL kinase domain [8, 9]. BCR-ABL Y177 in BCR domain synergistically promotes the occurrence of CML. Auto-phsphorylated BCR-ABL Y177 recruits the SH2 domain of adapter GRB2 (growth factor receptor bound protein 2), then GRB2 SH3 domains in N-terminal and C-terminal bind to GAB2 and SOS respectively, and activate corresponded RAS-MAPK and PI3K-AKT signal pathways, which promotes cell survival. BCR-ABL Y177F mutant defected in phosphorylation could not transform Rat-1 fibroblasts, and significantly reduce the pathogenecity in mice [10, 11]. Furthermore, previous work has demonstrated a kinase-independent mechanism of BCR-ABL Y177 in imatinib resistance [12]. These studies highlight the importance of phsphorylation of BCR-ABL Y177 in BCR-ABL oncogenecity and imply the potential of BCR-ABL Y177 as a logical therapeutic target of CML. In contrast to BCR-ABL and other protein tyrosine kinases (PTKs) phosphorylating substrates, protein tyrosine phosphatases (PTPs) function as an antagonist of PTKs and inhibit the phosphorylation of PTKs and/or their substrates [13, 14]. Protein tyrosine phosphatase 1B (PTP1B), one of the PTPs, has been reported as a tumor suppressor in CML cells by partly antagonizing BCR-ABL phosphorylation [15]. These studies raise the possibility that ectopic overexpression of PTP1B at BCR-ABL Y177 site could dephosphorylate BCR-ABL Y177 directly and forcibly, then uncouple the BCR-ABL Y177-GRB2 signal cascade, and inhibit BCR-ABL oncogenecity. It is reported that the catalytic domain of PTP1B (PTP1B/C) is necessary and sufficient to induce dephosphorylation [16, 17], so only the PTP1B/C is adopted in this work.

Besides phosphorylation, expression of BCR-ABL is the other precondition for its pathogenecity. Meanwhile, overexpression of BCR-ABL has been thought as one of the main mechanisms for imatinib resistance [18–20]. Great progress has been made to increase the ubiquitin-dependent degradation of BCR-ABL [21, 22], but this ubiquitin-dependent degradation process involving E1, E2 and E3 is too complex to be easily manipulated. Ornithine decarboxylase (ODC) is a classic ubiquitin-independent degradation protein, which is directed to proteasome for degradation without ubiquitination when binding to antizyme1 (AZ1) [23]. Because of the simplicity of ODC-AZ1 degradation system, researchers explored this system to degrade cellular proteins. For example, Matsuzawa fused the Rb binding domain of E7 with ODC, and coexpressed with AZ1. E7-ODC-AZ1 bound to Rb and directed it to proteasome for degradation [23]. This study indicates that if BCR-ABL was labeled with ODC, it may also be degraded by the protesome through ODC-AZ1 mediation.

Considering the importance of both phosphorylation and expression in BCR-ABL pathogenecity, we design a BCR-ABL dephosphorylation-degradation system named SPOA which is consisted of SPO fusion peptide and antizyme1. SPO fusion peptide is generated by fusing GRB2 SH2 domain, PTP1B/C, and ODC. SPOA binds to BCR-ABL Y177 through SH2, dephosphorylates BCR-ABL Y177 by PTP1B/C, and directs BCR-ABL to protesome for degradation by ODC-AZ1. Thus, SPOA uncouples downstream signal cascades triggered by phosphorylation of BCR-ABL Y177. Also, SPOA directs BCR-ABL to proteasome for degradation, therefore attenuates BCR-ABL downstream signal activation. In this work, we investigated whether SPOA dephosphorylated BCR-ABL Y177 and degraded BCR-ABL protein in ubiquitin-independent manner, and whether SPOA depressed the oncogenecity of BCR-ABL in imatinib sensitive and resistant CML cells and the underlying molecular mechanisms. We also evaluated the effect of SPOA on leukemogenesis in mice.

## RESULTS

### SPOA system dephosphorylates BCR-ABL Y177 in CML cells

**Figure 1:**
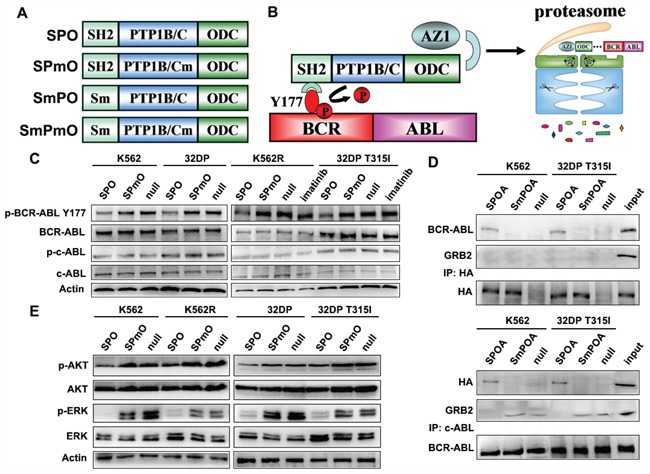
The dephosphorylation activity of SPOA system on BCR-ABL Y177 **A.** Schematic illustration of recombinant fusion peptides used in this study. **B.** The schematic diagram of SPOA system mediated dephosphorylation of BCR-ABL Y177 and degradation of BCR-ABL protein. **C.** Cells were treated with the indicated adenovirus or 3 μmol/L imatinib for 48 h. The phosphorylation of BCR-ABL Y177 and c-ABL Y245 were analyzed by western blot. **D.** The intracellular interaction between SPOA, BCR-ABL, and GRB2 was determined by co-immunoprecipitation assay. **E.** The effect of SPOA system on the GRB2 coupled signal cascade was analyzed by western blot. Figure shows the representative results of three replications.

GRB2 SH2 domain, PTP1B catalytic domain (PTP1B/C), and ornithine decarboxylase (ODC) were fused to generate the SPO fusion constructs with HA tag incorporated to the C-terminal. GRB2 SH2 R27 residue was substituted by lysine to generate a mutant, which failed to bind with BCR-ABL Y177 termed Sm [24]. PTP1B/C D181 residue was substituted by alanine to generate a mutant without catalytic activity termed PTP1B/Cm (Pm). Similar to SPO, SPmO, SmPO, and SmPmO were constructed by different combination of Sm, Pm, and ODC (Figure 1A). SPOA system consists of SPO fusion peptide and antizyme1 (AZ1). GRB2 SH2 domain directs PTP1B/C to BCR-ABL Y177 site and labels BCR-ABL with ODC (Figure 1B). To clarify whether SPOA system has dephosphorylation effect, we tested phosphorylated BCR-ABL Y177 (p-BCR-ABL Y177) in imatinib sensitive K562 and 32DP cell lines, and imatinib resistant K562R and 32DP T315I cell lines. As shown in Figure 1C, in contrast to imatinib inhibiting p-BCR-ABL Y177 only in imatinib sensitive cells, SPO dephosphorylated BCR-ABL Y177 in all cell lines, including the CML cells bearing T315I mutation. To survey the substrate specificity of SPO, we tested the phosphorylation of normal c-ABL kinase, a crucial regulator for cellular physiological function and the closest relative of BCR-ABL kinase, and found that it was not inhibited by SPO. These results demonstrate that SPOA system dephosphorylates BCR-ABL Y177 both in imatinib sensitive and resistant CML cells without decreasing c-ABL activation.

To investigate whether SPOA could bind to BCR-ABL Y177 intracellularly and dephosphorylate BCR-ABL Y177 to prevent it from recruiting GRB2, the interaction between SPOA, BCR-ABL, and GRB2 was determined by co-immunoprecipitation assay. As shown in Figure 1D, both in the precipitate of anti-HA antibody and anti-c-ABL antibody, the binding of BCR-ABL and SPOA, rather than SmPOA, was found. And in the precipitate of anti-c-ABL antibody, GRB2 was not detected only in SPOA group. These results indicate that SPOA could bind with BCR-ABL Y177 intracellularly, and prevent it from recruiting GRB2.

Auto-phosphorylation of BCR-ABL Y177 triggers RAS/MAPK and PI3K/AKT signals coupled by GRB2, which is involved in cell proliferation and survival. To observe whether SPOA impaired the signal cascade triggered by p-Y177, we analyzed the activation of ERK and AKT by western blot. As shown in Figure 1E, SPOA distinctly reduced the phosphorylation of ERK and AKT in imatinib sensitive and resistant CML cells. Taking together, these results demonstrate that SPOA interacts with and dephosphorylates BCR-ABL Y177, and prevents it from recruiting GRB2, which uncouples BCR-ABL Y177-GRB2 signal cascade.

### SPOA reduces BCR-ABL stability and directs BCR-ABL to proteasome for degradation by ubiquitin-independent pathway

**Figure 2:**
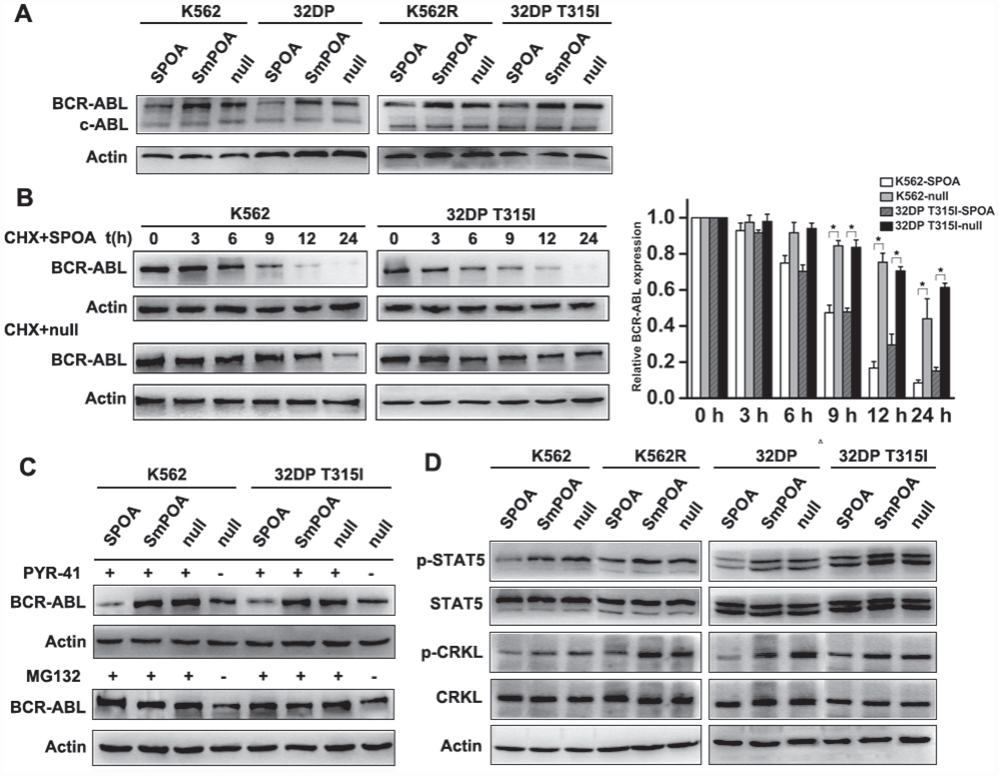
The degradative activity of SPOA system on BCR-ABL **A.** Cells were infected with the indicated adenovirus for 48 h, the expression of BCR-ABL and c-ABL were analyzed by western blot. **B.** Pretreated by SPOA for 48 h, the cells were treated with 20 mg/L cycloheximide (CHX) for another indicated time to inhibit BCR-ABL synthesis. BCR-ABL expression at the indicated time was determined by western blot (left). Gray values of protein bands were scanned with Quantity One® Version 4.5. The ratio of BCR-ABL to actin at 0 h was normalized to 1. The results were showed as fold change to 0 h (right). Data were represented as mean ± SD (n = 3). *p < 0.05 as compared to control. **C.** Pretreated with the indicated adenovirus for 36 h, the cells were treated with 20 μmol/L ubiquitin inhibitor PYR-41 or 20 μmol/L proteasome inhibitor MG132 for another 36 h. BCR-ABL expression was quantified by western blot. **D.** The effect of SPOA system on STAT5 and CRKL signal pathways were analyzed by western blot.

To directly observe the effect of SPOA on BCR-ABL expression, we assayed BCR-ABL expression in SPOA treated CML cells by western blot. As shown in Figure 2A, SPOA reduced BCR-ABL expression in imatinib sensitive and resistant CML cells without reducing c-ABL expression. To thoroughly evaluate the degradative effect of SPOA, BCR-ABL stability was analyzed. Protein synthesis was inhibited by cycloheximide. Changes of BCR-ABL expression over time were examined by western blot. We found that BCR-ABL expression in SPOA treated CML cells, either imatinib sensitive or resistant cells, began to sharp down at 9 h, and was almost undetectable at 24 h. In contrast, BCR-ABL expression in null group decreased much more slowly, either in K562 cells or in 32DP T315I cells (Figure 2B). These results demonstrate that SPOA reduces BCR-ABL stability and promotes BCR-ABL degradation in imatinib sensitive and resistant cells.

To clarify the degradative pattern of SPOA on BCR-ABL, ubiquitin inhibitor PYR-41 and proteasome inhibitor MG132 were employed. K562 and 32DP T315I cells were treated with SPOA and PYR-41 or MG132, then BCR-ABL expression was analyzed. As shown in Figure 2C, both PYR-41 and MG132 could inhibit spontaneous ubiquitin-dependent degradation of BCR-ABL and increase its expression. But, in SPOA treated CML cells, BCR-ABL degradation was not inhibited by PYR-41 while blocked by MG132. These results indicate that SPOA directs BCR-ABL to proteasome for degradation in ubiquitin-independent manner.

Besides GRB2 coupled RAS-MAPK and PI3K-AKT signal pathways, BCR-ABL also contributes to unregulated activation of several other signal molecules, such as STAT5 and CRKL, which synergistically participate in the pathogenesis of CML. As shown in Figure 2D, relatively low expression of p-STAT5 and p-CRKL were found in SPOA treated CML cells, which confirmed the degradative effect of SPOA on BCR-ABL in turn.

### SPOA specifically inhibits proliferation and promotes apoptosis of imatinib sensitive and resistant CML cells

**Figure 3:**
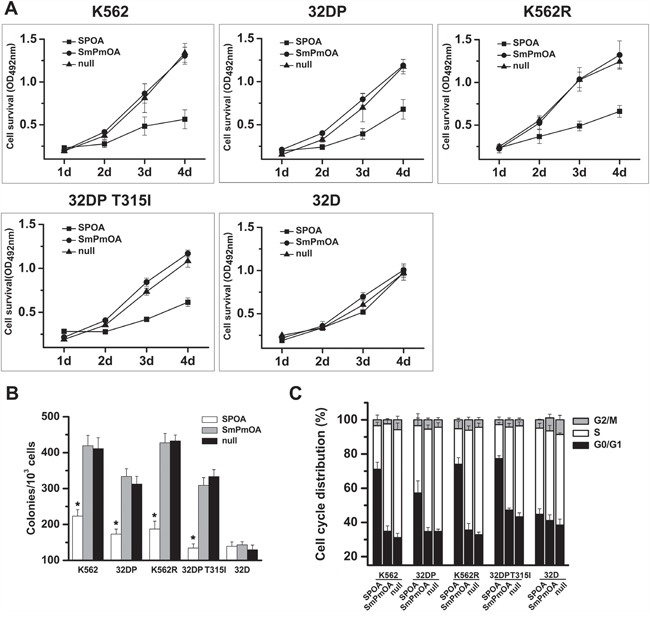
SPOA inhibits proliferation of imatinib sensitive and resistant CML cells **A.** Cells were plated in 96-well plates and infected with the indicated adenovirus. Cell viability was evaluated by the ability to transform MTT into purple formazan. Optical density at the wavelength of 492 nm (OD_492_) was measured at the indicated time. **B.** Cells were treated with the indicated adenovirus for 48 h, and plated in 24-well plates with methylcellulose for assessing the colony formation ability. Colonies were counted two weeks later using an inverted microscope. **C.** Cells were infected with the indicated adenovirus for 72 h, fixed in 70% pre-cooling ethanol, and incubated with propidium iodide for cell cycle analysis by a flow cytometer. All tests were carried out in triplicates. Data were represented as mean ± SD (n = 3). *p < 0.05 as compared to control.

Malignant proliferation is one of the main malignant phenotypes of CML cells [25]. The effect of SPOA on CML cell proliferation was evaluated by MTT assay and colony formation assay. We found that SPOA remarkably inhibited the growth (Figure 3A) and colony formation ability (Figure 3B and Supplementary Figure S1) of imatinib sensitive and resistant CML cells. To observe whether the anti-proliferation effect of SPOA is associated with cell cycle changes, we analyzed cell cycle distribution by flow cytometry. As shown in Figure 3C and Supplementary Figure S2, CML cell cycle in SPOA group was arrested in G0/G1 phase, which resulted in decreased division of CML cells. These results indicate that SPOA inhibits proliferation of imatinib sensitive and resistant CML cells through blocking cell cycle in G0/G1 phase.

**Figure 4:**
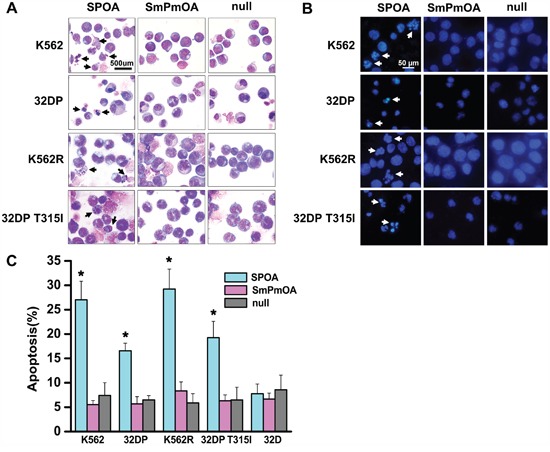
SPOA promotes apoptosis of imatinib sensitive and resistant CML cells Cells were infected with the indicated adenovirus for 72 h. Morphologic changes of apoptotic cells were visualized by Wright's staining **A.** (1000× magnification) and DAPI staining **B.** (400× magnification). For apoptotic rate analysis, cells were stained with AnnexinV-PE and 7-ADD, and determined by a flow cytometer **C.** All tests were carried out in triplicates. Data were represented as mean ± SD (n = 3). *p < 0.05 as compared to control.

Escaping from apoptosis is the other feature of CML cells [9]. To evaluate the effect of SPOA on the apoptosis of CML cells, Wright's staining and DAPI staining were performed to visualize the morphology changes of CML cells. As shown in Figure 4A and 4B, prominent apoptotic morphology, such as shriveled cell body, condensed or fragmented nuclear, and marginalized chromatin, were observed in SPOA treated CML cells. Furthermore, the apoptotic cell percentage in SPOA group increased significantly compared to control group as demonstrated by flow cytometry (Figure 4C and Supplementary Figure S3). These results confirm that SPOA induces apoptosis in imatinib sensitive and resistant CML cells.

We have confirmed that SPOA dephosphorylates BCR-ABL Y177, and degrades BCR-ABL protein without interfering the activation and expression of normal c-ABL. To fully elucidate the specificity of SPOA killing CML cells, we examined the effect of SPOA on the proliferation, cell cycle, and apoptosis of 32D cell line, which is a BCR-ABL negative myeloid cell line from C3H mice and the parental cell of 32DP and 32DP T315I. In accordance with the specificity of SPOA in molecular level, SPOA had no obvious cytotoxicity on 32D cells (Figure 3, Figure 4C, Supplementary Figure S1, Supplementary Figure S2 and Supplementary Figure S3). These results have proven that SPOA specifically inhibits proliferation and promotes apoptosis in CML cells.

### SPOA impairs the pathogenecity of K562 cells in mice

**Figure 5:**
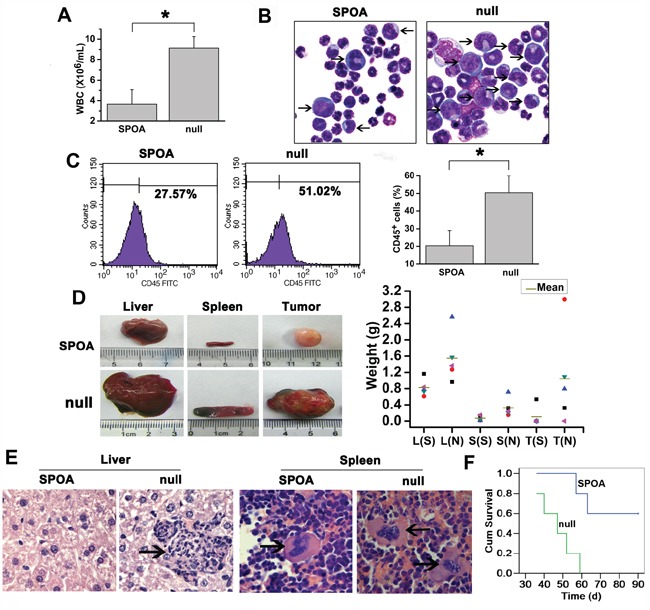
The anti-leukemia activity of SPOA in mice **A.** Comparison of the maximum of WBC count in SPOA group and null group. Data were represented as mean ± SD (n = 5). *p < 0.05 as compared to control. **B.** Leukemic blasts in bone marrow of diseased mice were analyzed by Wright's staining (1000× magnification). The arrows indicate the immature or blast cells. **C.** Human CD45^+^ cells in murine bone marrow were counted by flow cytometry. Data were represented as mean ± SD (n = 5). *p < 0.05 as compared to control. **D.** Comparison of the size (left) and weight (right) of liver (L), spleen (S), and solid tumor (T) in SPOA group (S) and null group (N). Without solid tumor was regarded as weight=0 g. **E.** The leukemic infiltration of liver and spleen was analyzed by HE staining (1000× magnification). The arrows indicate the infiltrated leukemic cell clusters. **F.** Kaplan-Meier survival analysis of mice.

Our studies above have shown that SPOA inhibits leukemogenesis *in vitro*. To evaluate the effect of SPOA on leukemogenesis in mice, K562 cells pretreated with SPOA or null adenovirus were injected intravenously into NOD-SCID mice to develop the CML-like disease. Weight, leukocyte count, and life daily of mice were monitored regularly. Four weeks later, the symptoms of CML-like disease in mice including weight loss, leukocytes increase, hind-limb paralysis, fluffy hair, and reduced activity [9], appeared first in null group. Leukocytes both in SPOA group and null group increased gradually, and reached the maximum around the time of getting disease. The maximum of leukocyte count in SPOA group was significantly lower than that of null group (*P<0.05, Figure 5A). By Wright's staining of peripheral blood (Supplementary Figure S4) and bone marrow smears (Figure 5B), primitive and immature cells were more in null group. To understand the relative contribution of the individual cell types, we analyzed murine hemogram and myelogram by cytological classification. In peripheral blood, granulocytes account for more than 90% of the leukocytes both in SPOA group and null group, but the immature and blast cells were significantly more in null group (p<0.05) (SPOA: 5%±2%, null: 29%±8%). In bone marrow, there were no significant difference in the distribution of each series between SPOA group (granulocyte: 78%±7%, erythroid: 15%±2%) and null group (granulocyte: 81%±9%, erythroid: 12%±4%) (p>0.05). But, the immature and blast cells were significantly more in null group than SPOA group (p<0.05) (SPOA: 33%±7%, null: 69%±9%). The other series in both group, such as monocytes and lymphocytes, were almost absent in both peripheral blood and bone marrow. To explore whether the cells were leukemic, we counted human CD45^+^ cells by flow cytometry, which reflect the number of K562 cells in murine bone marrow. As shown in Figure 5C, CD45^+^ cells were significantly less in SPOA group than null group (*p<0.05). These results indicate that SPOA significantly inhibits the proliferation of K562 cells in NOD-SCID mice. Furthermore, we observed more severe hepatomegaly and splenomegaly in null group compared to SPOA group (P<0.05) (liver: 1.55±0.61g for null group and 0.82±0.21g for SPOA group; spleen: 0.33 ± 0.23 g for null group and 0.07 ± 0.06 g for SPOA group). In addition, solid tumors in enterocoelia were bigger and more common in null group (Figure 5D). Consistently, more severe leukemic infiltration and tissue destruction of liver and spleen were found in null group by HE staining (Figure 5E and Supplementary Figure S5). At the end of the murine experiments for 90 days, all the mice (5 of 5) in null group developed the CML-like disease, compared to 2 of 5 in SPOA group. The disease latency in null group was 38.4 ± 7.8 d. In contrast, latency of the only two diseased mice in SPOA group was 49 days and 54 days. These results indicate that SPOA could prolong the disease latency in mice. By Kaplan-Meier survival analysis, we found that SPOA significantly prolonged mice survival time (p<0.05), with three mice surviving free of disease (Figure 5F). Taken together, these results confirm a significant anti-leukemogenesis role of SPOA in mice.

## DISCUSSION

BCR-ABL oncoprotein with constitutively activated tyrosine kinase activity is responsible for the pathogenesis of CML. Imatinib has shown potent activity in chronic CML patients, which inhibits BCR-ABL tyrosine kinase activity and controls the progression of CML [26, 27]. However, imatinib resistance due to mutations in BCR-ABL kinase domain is rapidly acquired during therapy in many patients [28, 29]. So, great efforts have been made to develop more potent second generation of TKIs, such as dasatinib and nilotinib, which succeed in circumventing resistance caused by most of the mutations except the gatekeeper T315I mutation [30]. In particular, ponatinib, one of the third generation of TKIs, shows great activity against T315I mutation, and has been approved for the therapy of CML patients with T315I mutation [31]. Unfortunately, ponatinib was suspended for sale because of severe side effects [32, 33]. In fact, besides BCR-ABL, some other kinases are simultaneously inhibited by ponatinib, such as Aurora kinase, FLT3, c-KIT, c-ABL, and SRC, which may partly explain the cause of the side effects [34, 35]. Hence, more effective therapies are needed to overcome drug resistance.

Several domains of BCR-ABL protein synergistically participate in the pathogenesis of CML [36]. Imperfect effect by targeting ABL domain allows us to focus on Y177 in BCR domain. The autophosphorylation of BCR-ABL Y177 has been considered as the initial event of activation of GRB2 coupled RAS-MAPK and PI3K-AKT signal pathways, which promotes cell proliferation and survival [37]. Previous work has found that BCR-ABL Y177F mutant, defecting in phosphorylation, could not transform Rat1 fibroblast cells [10], implying that inhibition of BCR-ABL Y177 phosphorylation is expected to inactivate BCR-ABL and depress its transformation ability. Besides the mutations in BCR-ABL kinase domain, overexpression of BCR-ABL protein, resulted from amplification of bcr-abl gene, has been thought as the other predominant factor of imatinib resistance [18]. Comprehensively, a strategy inactivating and degrading BCR-ABL protein is likely to override imatinib resistance. In this study, we designed a dephosphorylation and degradation system targeting BCR-ABL Y177 termed SPOA, and tested its anti-leukemia activity *in vitro* and *in vivo*. We showed that, SPOA potently dephosphorylated BCR-ABL Y177 both in imatinib sensitive and resistant CML cells. Also, SPOA distinctly degraded BCR-ABL protein, which was not inhibited by the ubiquitin inhibitor PYR-41, but blocked by the proteasome inhibitor MG132, indicating the ubiquitin-independent ODC-AZ1 degradation manner. As a result, the dephosphorylation and degradation activity of SPOA on BCR-ABL synergistically inhibited proliferation and promoted apoptosis in imatinib sensitive and resistant CML cells. Interestingly, SPOA did not reduce the activation and expression of normal c-ABL kinase, and had no cytotoxicity on BCR-ABL negative cells, which partly confirmed the specificity of SPOA targeting CML cells.

BCR-ABL aberrantly activates multiple signal pathways involving leukemic cell proliferation and survival. Besides GRB2 coupled RAS-MAPK and PI3K-AKT signal pathways, BCR-ABL also activates STAT5 and CRKL signal molecules [38–40]. To elucidate the exact anti-leukemia mechanism of SPOA, we tested the effect of SPOA on the binding of BCR-ABL with GRB2, and the signal activation by BCR-ABL. As expected, the binding of SPOA and BCR-ABL prevented BCR-ABL from recruiting GRB2, and reduced multiple signal activity, including RAS-MAPK, PI3K-AKT, STAT5, and CRKL, in imatinib sensitive and resistant CML cells. These results reveal that the dephosphorylation and degradation of BCR-ABL by SPOA inhibit the signal transduction of BCR-ABL, which plays the anti-leukemia effect in CML cells, even with highly resistant T315I mutation. Also, we found that SPOA impaired leukemogenesis of K562 cells in mice. SPOA significantly inhibited the proliferation of K562 cells in mice, and reduced leukemic infiltration. Consequently, SPOA prolonged the disease latency, reduced the incidence, and improved survival in mice. Notably, two of five mice in SPOA group also developed CML-like disease even though at a longer latency. This imperfect effect should be optimized by improved delivery methods.

In conclusion, our results provide evidence that SPOA has potent anti-leukemia activity *in vitro* and *in vivo* by specifically dephosphorylating and degrading BCR-ABL oncoprotein. This novel strategy may provide alternative therapy for CML patients, especially with imatinib resistance. For potentially clinical applications, hematopoietic stem cells should be removed from CML patients, treated with efficient delivery vector expressing SPOA, and amplified for autotransfusion.

## MATERIALS AND METHODS

### Reagents and antibodies

Ubiquitin inhibitor PYR-41 (Sigma-Aldrich, USA), proteasome inhibitor MG132 (Sigma-Aldrich, USA), protein synthesis inhibitor cycloheximide (Sigma-Aldrich, USA), and imatinib (Novartis Pharmaceuticals, USA) were all diluted in DMSO. Antibodies of phosphorylated BCR Y177, c-ABL Y245, STAT5 Y694, AKT T308, ERK1/2 T202 and Y204, CRKL Y207, and antibodies of total c-ABL, STAT5, AKT, ERK1/2, CRKL, GRB2, and HA were all purchased from Cell Signaling Technology (USA). Anti-β-Actin antibody was purchased from Santa Cruz Biotechnology (USA). Pierce™ HA-Tag IP/Co-IP Kit and Pierce™ Classic Magnetic IP/Co-IP Kit were purchased from Thermo Scientific (USA).

### Cell culture

Cells were maintained in RPMI 1640 contained of 10% FBS (Gibco, USA) and 2 mmol/L L-glutamine. For 32D cells, additional 700 ng/L IL-3 was supplemented. K562R cells were generated from K562 cells screened with imatinib to develop imatinib resistance as described previously [41]. 32DP and 32DP T315I cells were generated from 32D cells which were stably transformed by p210^BCR-ABL^ or p210^BCR-ABL^ bearing T315I mutation.

### Constructs

The gene encoding SH2, PTP1B/C, ODC, or AZ1 was amplified through PCR from human cDNA. SH2 R27K (Sm) and PTP1B/C D181A (Pm) mutants were generated by overlapping PCR. SH2, PTP1B/C, and ODC were stepwise inserted into pAdTrack-CMV vector at the Kpn I, Sal I, Not I, and Xba I sites to develop a fusion gene termed SPO. SPmO, SmPO, and SmPmO were constructed similarly. AZ1 was inserted into pAdTrack-CMV vector at the Kpn I and Sal I sites. HA or FLAG tag was respectively added to the carboxyl terminal of ODC or AZ1. The expression cassette was used to generate recombinant adenovirus using the AdEasy system as previously described [42]. Cells were infected at an MOI of 10^5^:1 for K562 and K562R cells or 3 × 10^5^:1 for 32D, 32DP and 32DP T315I cells.

### Western blot

Proteins were resolved by 6%~12% SDS-PAGE and transferred to PVDF membranes. The membranes were blocked with 5% nonfat milk in TBST, and incubated with primary antibodies overnight. After washing, the membranes were incubated with HRP-conjugated secondary antibodies. Protein bands were visualized by enhanced chemiluminescence on cool image workstation II (Viagene, USA). The expression of β-Actin was for control.

### Co-immunoprecipitation assay

Cells were treated with each adenovirus for 48 h, and collected for Co-immunoprecipitation assay. All assays were performed according to the manufacturer's instructions of Pierce™ HA-Tag IP/Co-IP Kit and Pierce™ Classic Magnetic IP/Co-IP Kit (Thermo Scientific, USA). In brief, for anti-HA immunoprecipitation assay, equal protein lysates were precipitated with anti-HA agarose beads at 4°C; overnight. For anti-c-ABL immunoprecipitation assay, equal protein lysates were precipitated with anti-c-ABL antibody at 4°C; overnight, the mixture was incubated with free Protein A/G agarose beads for another one hour. The co-immunoprecipitate was followed by western blot.

### MTT assay

Cell growth was analyzed by MTT assay. Cells were plated in 96-well plates at a density of 2 × 10^3^ cells per well with three duplications for each treatment. Cell viability was evaluated by the ability to transform 3-(4, 5-dimethylthiazol-2-yl)-2, 5-diphenyltetrazolium bromide (MTT) (Sigma, USA) into purple formazan. Optical density at the wavelength of 492 nm (OD_492_) was measured on a microplate reader at the indicated time.

### Colony formation assay

Cells were treated with adenovirus for 48 h, and plated in 24-well plates with methylcellulose at a density of 500 cells per well for assessing the colony formation ability. Colonies were counted two weeks later using an inverted microscope (Olympus, Japan). All analyses were carried out in triplicates.

### Morphological visualization of apoptosis

Cells were treated with adenovirus for 72 h. The cell smears were fixed and stained with Wright's stain or DAPI stain. The apoptotic morphology was observed under microscope.

### Flow cytometric analysis of cell cycle and apoptosis

Cells were infected with adenovirus for 72 h. Cell cycle and apoptosis were analyzed by flow cytometry. In brief, for apoptotic analysis, cells were stained with AnnexinV-PE and 7-ADD (KeyGenBiotech, China) following manufacturer's instructions. For cell cycle analysis, cells were fixed in 70% pre-cooling ethanol, and incubated with propidium iodide (PI) (Sigma, USA). Cell cycle and apoptosis were analyzed by a Beckman Coulter EPICS Elite flow cytometer. All tests were carried out in triplicates.

### Murine BCR-ABL leukemogenesis assay

All animal experiments were approved by the Institutional Animal Care and Use Committee of Chongqing Medical University, and conducted strictly in accordance with relevant protocols. Six week old female NOD-SCID mice were randomized into two groups (n=5 for each group). K562 cells were pretreated with SPOA or null adenovirus for 72 h, washed with PBS for twice, and resuspended in PBS. 5×10^6^ cells were injected intravenously into sublethally irradiated mice. Weight, leukocyte count, and life daily of mice were monitored regularly.

### Analysis of diseased mice

Weight loss, leukocyte increase, hind-limb paralysis, fluffy hair, and reduced activity were regarded as the symptoms of CML-like disease in mice. Mice in articulo mortis were sacrificed. Proliferation and organ infiltration of leukemia cells in mice were evaluated by Wright's staining, flow cytometry, and HE staining. In detail, peripheral blood and bone marrow smears were stained with Wright's stain to observe the leukemic blasts. Relative contribution of the individual cell types were evaluated by cytological classification of 100 nucleated cells. Marrow cells were harvested, incubated with antibody to human CD45, and analyzed for the rate of CD45^+^ cells by flow cytometry. HE staining was performed using paraffin sections of liver and spleen tissues as previously described [9].

### Statistical analysis

All data were repeated at least thrice. Data were presented as mean±SD. Statistical analysis was performed using Student's t test. The value P < 0.05 was assigned as statistically significant. Kaplan-Meier survival curves were graphed using SPSS 13.0.

## SUPPLEMENTARY MATERIALS FIGURES


