# Supplementary material for: Depression of oncogenecity by dephosphorylating and degrading BCR-ABL
Source: Oncotarget. 2016 Dec 1;8(2):3304–14. doi: 10.18632/oncotarget.13754 (PMC5356883; doi:10.18632/oncotarget.13754)
Supplement: Supplementary file 1 [file oncotarget-08-3304-s001.pdf]

Depression of oncogenecity by dephosphorylating and degrading BCR-ABL

SUPPLEMENTARY FIGURES

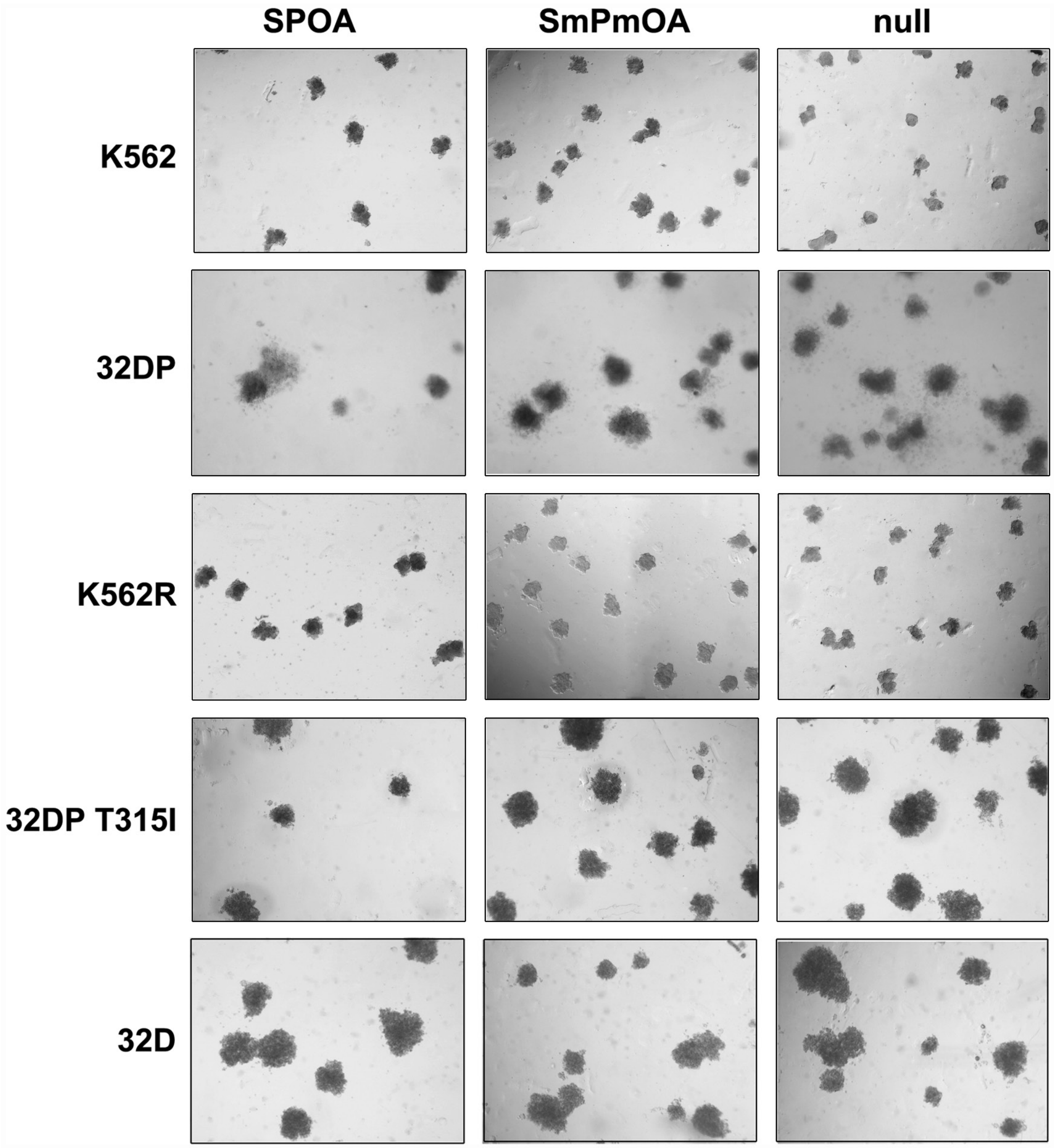

Supplementary Figure S1: Inhibitory effect of SPOA on the colony formation ability of CML cells. Cells were infected with the indicated adenovirus for 48 h and plated in 24-well plates with methylcellulose. Cell proliferation was evaluated by colony formation assay. Colonies were visualized under microscopy (40× magnification) two weeks later. The experiment was repeated thrice. Figure shows the representative results.

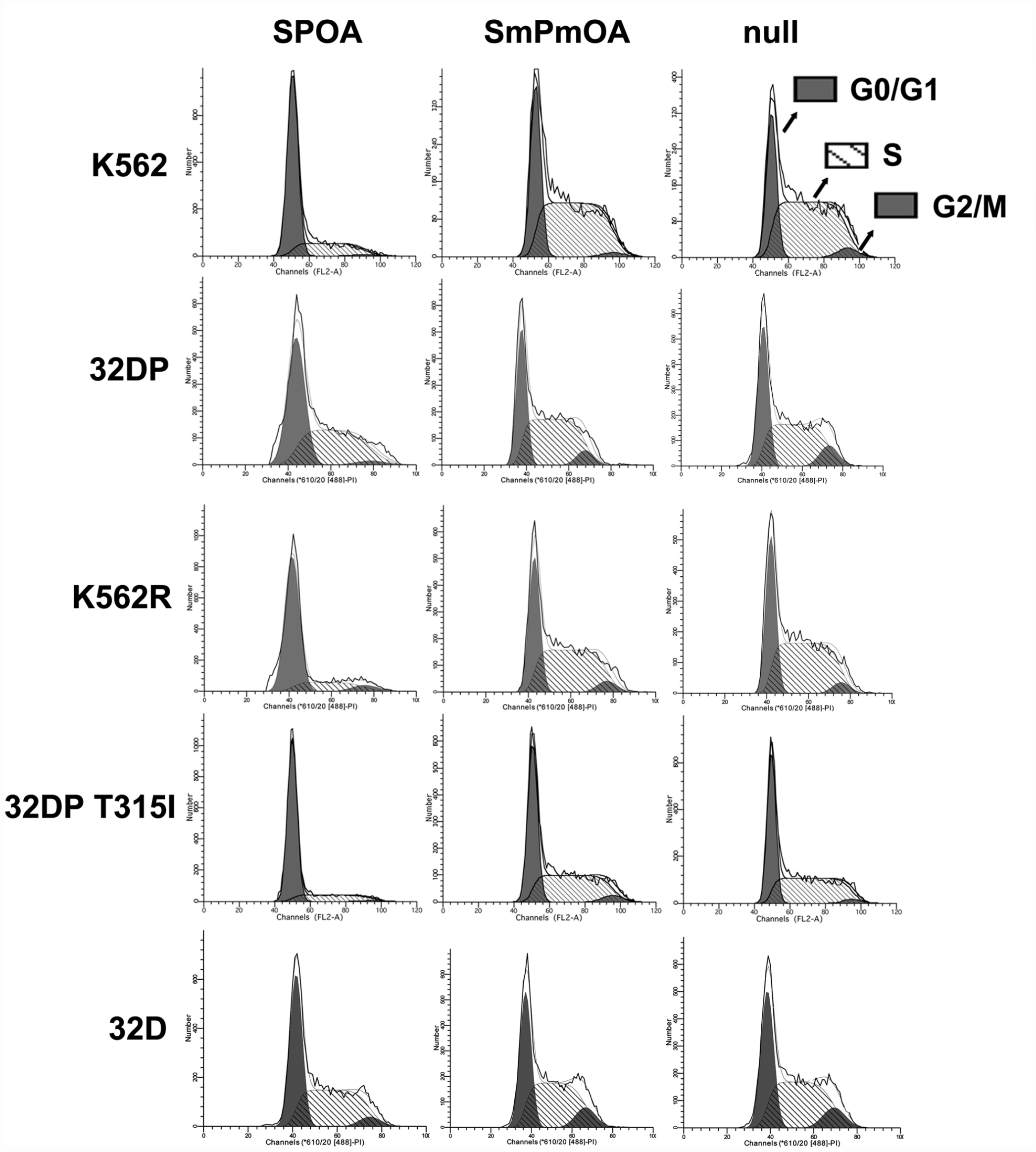

Supplementary Figure S2: Blocking effect of SPOA on CML cell cycle. Cells were infected with the indicated adenovirus for 72 h, fixed in 70% pre-cooling ethanol, and incubated with propidium iodide. Cell cycle was analyzed by flow cytometry. The experiment was repeated thrice. Figure shows the representative results.

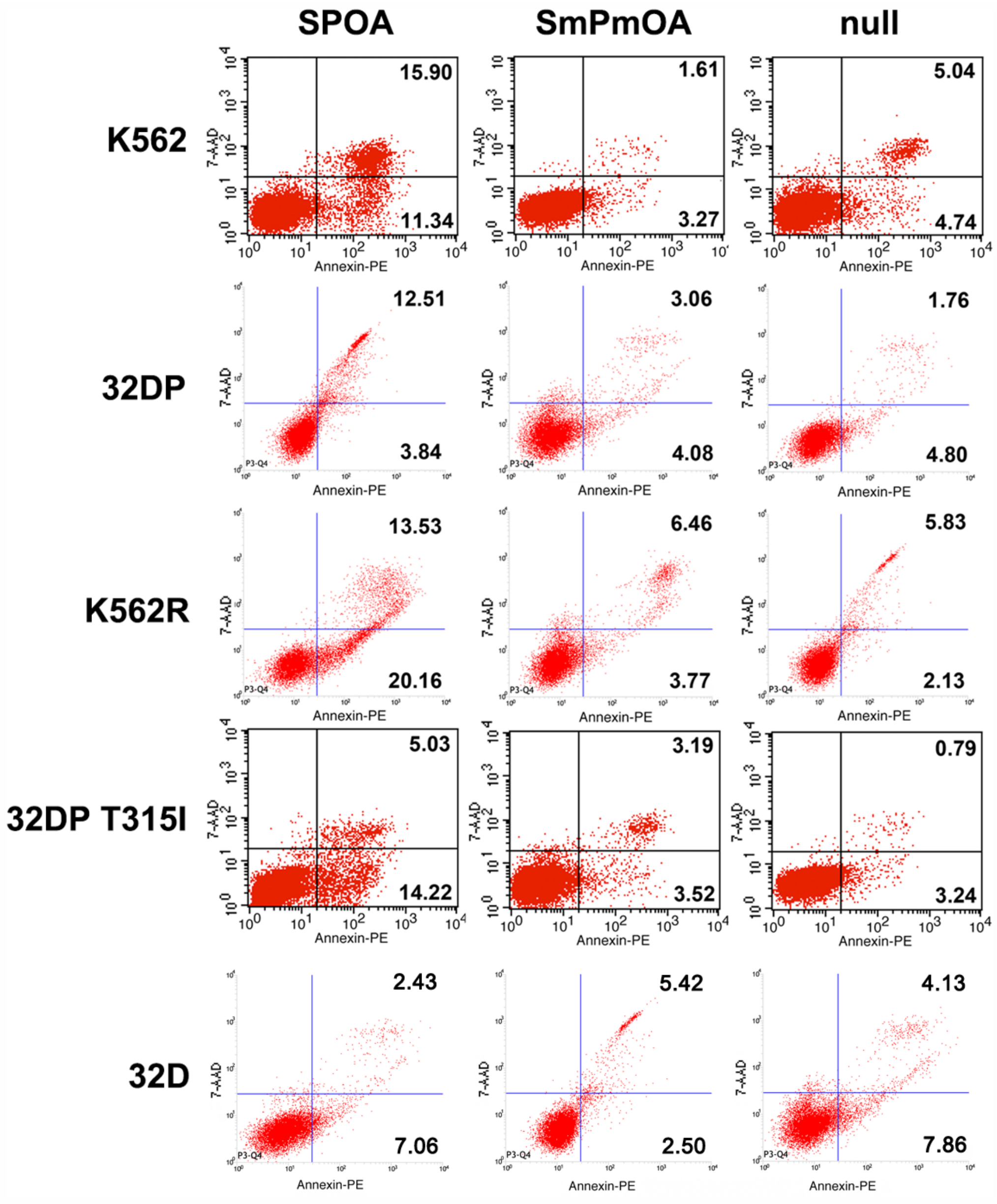

Supplementary Figure S3: Proapoptosis effect of SPOA on CML cells. Cells were infected with the indicated adenovirus for 72 h, stained with AnnexinV-PE and 7-ADD. Cell apoptosis was analyzed by flow cytometry. The experiment was repeated thrice. Figure shows the representative results.

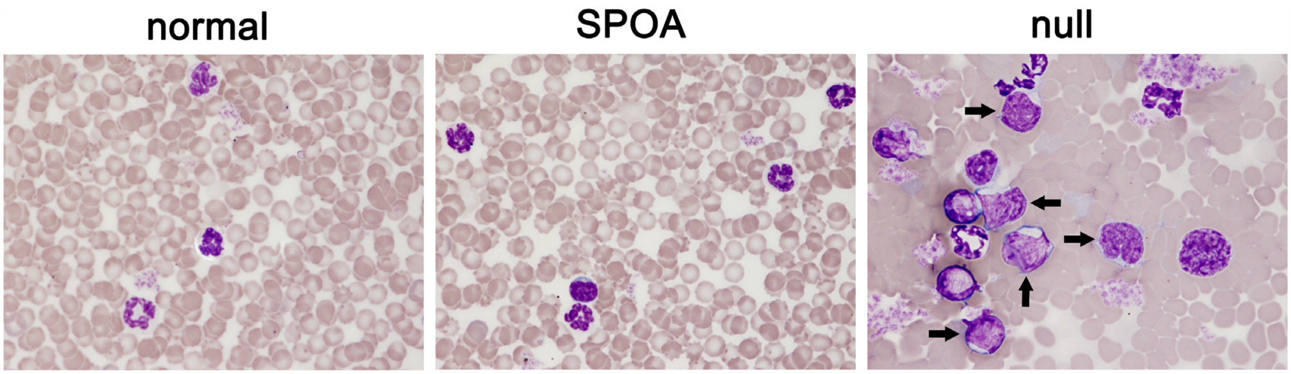

Supplementary Figure S4: Increased leukemic blasts in peripheral blood of diseased mice. Leukemic blasts in peripheral blood of diseased mice were evaluated by Wright's staining (1000 × magnification). The arrows indicate the immature or blast cells. Figure shows the representative results.

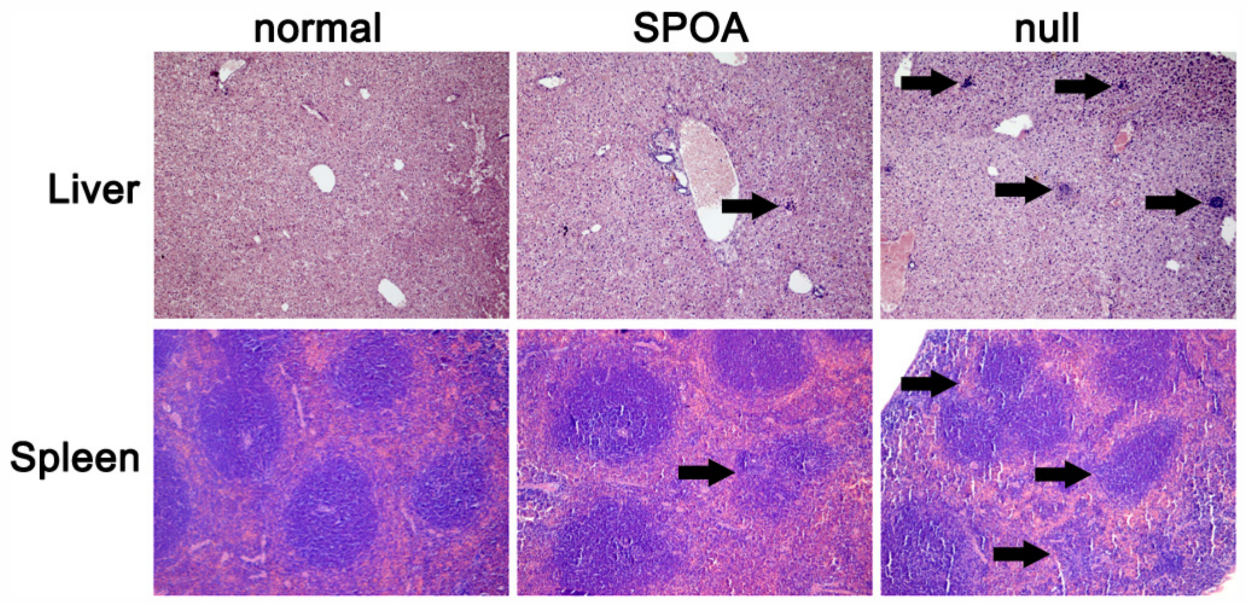

Supplementary Figure S5: Leukemic infiltration in liver and spleen of diseased mice. Leukemic infiltration in liver and spleen of diseased mice were analyzed by HE staining (100 × magnification). Arrows indicate the infiltrated leukemic cell clusters in liver and the destroyed splenic nodules. Figure shows the representative results.
